# SIRT1 and SIRT2 Modulators: Potential Anti-Inflammatory Treatment for Depression?

**DOI:** 10.3390/biom11030353

**Published:** 2021-02-25

**Authors:** Yuqing Zhang, Shailendra Anoopkumar-Dukie, Andrew Keith Davey

**Affiliations:** 1 Menzies Health Institute Queensland, Griffith University, Gold Coast, QLD 4222, Australia; yuqing.zhang3@griffithuni.edu.au (Y.Z.); s.dukie@griffith.edu.au (S.A.-D.); 2 School of Pharmacy and Pharmacology, Griffith University, Gold Coast, QLD 4222, Australia; 3 Quality Use of Medicines Network, Griffith University, Gold Coast, QLD 4222, Australia

**Keywords:** depression, inflammation, SIRT1, SIRT2, clinical study, polymorphism

## Abstract

Depression is a psychiatric disorder that has a significant health burden on patients and their families. Unfortunately, the current antidepressant medications that mainly target monoamine neurotransmitters have limited efficacy. Recent evidence has indicated that neuroinflammation participates in the genesis and development of depression, and interacts with other factors involved in depression. Therefore, exploring effective anti-inflammatory medications could be beneficial for the development of new treatment options for depression. Sirtuins are a unique class of nicotinamide adenine dinucleotide (NAD^+^)-dependent deacetylases, which have seven members that can affect multiple downstream targets by deacetylation activity. Among these seven members, SIRT1 and SIRT2 have been shown to participate in the pathophysiology of inflammation in numerous studies. Thus, in this short article, we review the association of SIRT1 and SIRT2 activity and depression, and evidence of the effects of SIRT1 and SIRT2 modulators on inflammation in vitro and depressive-like behaviours in vivo.

## 1. Background

Depression is one of the leading causes of disability [1]. According to the data published by the World Health Organisation, over 264 million people of all ages suffer from depression, and approximately 800,000 people die from suicide every year [2]. The main theories of the underlying pathophysiology of depression include deficiency of monoamine neurotransmitters such as serotonin, noradrenaline and dopamine in the central nervous system, dysregulation of the hypothalamic–pituitary–adrenal (HPA) axis, and reduction in brain-derived neurotrophic factor (BDNF). However, there is strong evidence suggesting an intimate linkage between chronic neuroinflammation and the development of depression and associated somatic manifestations, such as disturbed sleep and a loss of appetite [3,4,5]. There are no apparent infectious insults in the initiation of depression, but depressed patients without physical diseases do show an increased level of inflammatory markers [6]. TNF-α, IL-6 and C-reactive protein are all higher than normal in major depressive disorders [7]. Excessive inflammatory cytokines can influence other mechanisms of depression by decreasing the production of serotonin and BDNF, fueling glutamate excitotoxicity and disrupting the HPA axis and hormone balance [8]. However, there are no medication options that target the inflammatory process applied in the clinical treatment of depression.

### 1.1. Current Medication Treatments for Depression and Their Limitations

Current commercially available antidepressants are designed to increase levels of monoamine neurotransmitters via different mechanisms [9]. First-generation antidepressants, including tricyclic antidepressants (TCAs) and monoamine oxidase inhibitors (MAOIs), are frequently accompanied by undesirable side-effects and toxic effects in overdose, limiting their application [9]. Newer-generation antidepressants, including the selective serotonin reuptake inhibitors (SSRIs), are more selective and offer improved safety and tolerability. However, their therapeutic efficacy is still limited and can be delayed in depression patients without lower levels of neurotransmitters [9].

The National Institute of Mental Health (NIMH)-funded sequenced treatment alternatives to relieve depression (STAR*D) study was conducted to assess the effectiveness of various antidepressants for people with major depression who had not responded to initial treatment with a current antidepressant [10]. The overall remission rates for the present medication options, including citalopram, bupropion, venlafaxine, sertraline and mirtazapine, were less than 30% [11,12,13,14,15,16,17,18]. In addition, even after four sequential pharmacotherapies, 19% of patients still had disease that was resistant to treatment [19].

### 1.2. SIRT1 and SIRT2’s Role in Inflammation

The sirtuin family is a unique class of nicotinamide adenine dinucleotide (NAD^+^)-dependent deacetylases. There are seven enzyme members that differ in their subcellular localization, enzymatic activities, physiological functions and pathological roles. Sirtuins accomplish their primary enzymatic activities through NAD^+^-nicotinamide exchange reaction, in which lysines on histones are catalyzed through deacetylation [20,21]. Two important members, SIRT1 and SIRT2, have been found to participate in the pathophysiology of depression. SIRT1 has several potential roles in depression through effects on inflammation, neurogenesis, circadian rhythm and other stress-related extracellular signal-regulated kinase pathways [22]. Importantly, inflammation appears to be either a vital mediator or an important downstream effect of these various contributory factors [23]. Furthermore, SIRT1 has been considered to suppress inflammatory gene expression via the deacetylation in post-translational modification and associated transcription factors such as nuclear histone, NF-κB, activator protein-1, tumor suppressor p53 and peroxisome proliferator-activated receptor-γ (PPARγ) [24]. SIRT1 activation can also exert protection in neurons by improving mitochondrial biogenesis and antagonizing apoptosis via multiple mechanisms, which might augment SIRT1’s potential anti-inflammatory effects [25]. Current evidence suggests that SIRT2 exerts the opposite effect to SIRT1 in neurological studies [26]. SIRT2 also has been reported to be involved in inflammation-related conditions, such as sepsis, brain injury and insulin resistance [27,28,29]. However, whether the role of SIRT2 in inflammation is related to suppression or protection remains controversial. 

SIRT1 and SIRT2 modulators have been studied in numerous conditions including cancer, diabetes, cardiovascular and neurodegenerative diseases, and have been found to have a positive effect on cell survival and longevity [30,31,32,33]. Although very limited data can be obtained from existing clinical trials of SIRT modulators in depression, some evidence supporting the anti-inflammatory effects of the SIRT1 activators can be found in clinical trials of other inflammation-associated conditions. SRT2104 is a selective SIRT1 activator assessed in multiple trials. A randomized, double-blind, placebo-controlled study in healthy subjects (*n* = 24) was conducted to assess the anti-inflammatory effect of SRT2104 pretreatment against acute stimulation by LPS intravenous injection [34]. The results showed that there was a reduction in IL-6 and IL-8 and the plasma level of C reactive protein by SRT2104 oral administration with single (2 g) or multiple doses (2 g/d for 7 days) [34]. However, the levels of TNF-α and IL-10 were not significantly altered by SRT2104, and IL-1β was not detectable in LPS-induced subjects [34]. Meanwhile, leucocyte counts and gene expression remained unaltered with pre-treatment of SRT2104, which indicates that SRT2104 did not affect the activities of intravascular leukocytes 4 h after LPS stimulation and extravascular mechanisms may be involved [34]. In a randomized, double-blind, placebo-controlled, phase IIa clinical trial of psoriasis (*n* = 40), subjects were administered SRT2104 capsules with doses of 250, 500 or 1000 mg/day for eighty-four days [35]. The outcome of skin biopsies showed that SRT2104 significantly reduced the epidermal thickness to a normal level, normalized keratinocyte differentiation and suppressed the expression of keratinocyte hyperproliferation gene marker [35]. Furthermore, the expression of inflammatory cytokine (TNF-α and IL-17)-responsive genes, including serine protease inhibitors and L-kynureninase (KYNU), was also shown to be reduced by SRT2104 treatment [35].

There is a growing body of evidence that SIRT1 and SIRT2 may have a role in depression, linked to inflammation. As such, these studies provide an early indication that there may be a role for SIRT1 and SIRT2 modulators as an adjunct to current antidepressant therapy regimes. This review will discuss the evidence to date in relation to the purported role of SIRT1 and SIRT2 modulators in the treatment of depression. 

## 2. Association of SIRT1, SIRT2 Polymorphisms and Depression 

The etiology of depression has been extensively studied in relation to pathophysiologic activities, such as a deficiency of monoamine neurotransmitters, HPA axis dysregulation and inflammation, but genetic influences also need to be considered [36,37]. For instance, polymorphisms in key genes encoding serotonin transporters, hormone receptors, neurotrophin protein and cytokines lead to alterations in transcription and, subsequently, a lower or higher expression of these functional proteins [37]. 

Furthermore, polymorphisms or genetic variation in SIRT1 and SIRT2 in the normal and depression populations have also been studied to investigate the genetic association of SIRT1 and SIRT2 and the susceptibility, onset and course of depression. A genome-wide association study (GWAS) with more than 9000 cases conducted on Chinese women with major depressive disorder (MDD) identified that single-nucleotide polymorphism (SNP) rs12415800, a gene locus near SIRT1 on chromosome 10, might contribute to the risk of MDD [38]. Another GWAS study carried on the Chinese Han population has investigated the subsequent effect of the risk allele rs12415800 [39]. It has been found that rs12415800 is associated with the reduction in cerebellar grey matter volume and lower SIRT1 mRNA expression in amygdala tissue, contributing to the abnormal emotion processing in MDD [39]. This SNP, along with rs4746720, which is located in the 3′-untranslated region of SIRT1, were both found to have a significant association with suicide in Japanese women (778 suicide completers and 760 controls) [40]. This study also examined the SIRT1 mRNA expression only in the dorsolateral prefrontal cortex and found no association between the expression and suicide [40]. Besides, a case-control study of SIRT1 SNPs and major depressive disorder patients (455 MDD patients and 766 controls) in the Japanese population also suggested a significant association between SIRT1 rs10997875 polymorphism and depression [41]. SIRT2 gene rs10410544 polymorphism has also been reported to associate with depressive symptomatology in Alzheimer’s disease in two independent European populations [42]. It was also indicated that the SIRT2 T/T genotype might exert protection against depression [42]. Another case study of SIRT polymorphisms and postpartum depressive symptoms (PDSs) identified significant correlations between SIRT2 SNPs at rs2873703 (T/T) and rs4801933 (T/T) and the occurrence of PDSs [43]. Nonetheless, more studies are needed to verify the effects of these SNPs on the expression and biological function of SIRT1 and SIRT2. A summary of these polymorphism studies is shown in Table 1.

## 3. SIRT1 and SIRT2 Modulators in In Vitro Studies of Inflammation

While the direct relationship between inflammatory mediators and the pathology of depression has not been fully elucidated, the evidence of the elevation of cytokines in depression provided by clinical studies is considerable [44]. However, high variations were found in the serum level of the inflammatory mediators in depression patients [44]. Thus, it is not feasible to clearly define the characteristics that an in vitro model should have to accurately represent the inflammatory components of depression. Hence, the evidence of the anti-inflammatory effects of SIRT1 and SIRT2 modulators can be found in studies applying different neurological in vitro models. 

For SIRT1 activators, resveratrol has been proven to have anti-inflammatory effects in multiple neurological disease models including LPS-induced microglia and neurotoxin-induced models of Parkinson’s or Alzheimer’s disease [45]. SRT2104 is a well-tolerated SIRT1 activator, which has already been assessed in clinical trials [46]. Its anti-inflammatory effects have been tested in psoriasis clinical trials and studies of LPS-injected healthy human subjects, as discussed above. However, there have been no reported in vitro studies investigating SRT2104’s effects on neuroinflammation to date. Regarding SIRT2 inhibitors, SIRT2 is often reported to exert the opposing effect to SIRT1 in neurodegeneration studies [47]. However, based on available in vitro studies of inflammation, different reports using various in vitro models and stimuli have reported contradictory findings, suggesting that the effect of SIRT2 modulators needs to be interpreted based on the biochemical conditions of different models. For example, an in vitro study of brain injury showed that the SIRT2 inhibitor AK-7 can increase the expression of pro-inflammatory cytokines in primary microglia after stretch-induced injury [48], and a similar pro-inflammatory effect of AK7 was reported in a cell culture system that investigated spinal cord injury [49]. However, in a study of allergic asthmatic inflammation, SIRT2 inhibitor AGK2 diminished inflammatory manifestations, including the activation of lung macrophages and expression of chemokine CCL17 [50]. Sirtinol, as a dual inhibitor of SIRT1 and SIRT2, was also shown to reduce inflammation in primary dermal microvascular endothelial cells [51].

## 4. SIRT1 and SIRT2 Modulators in In Vivo Studies of Depression

**Figure 1:**
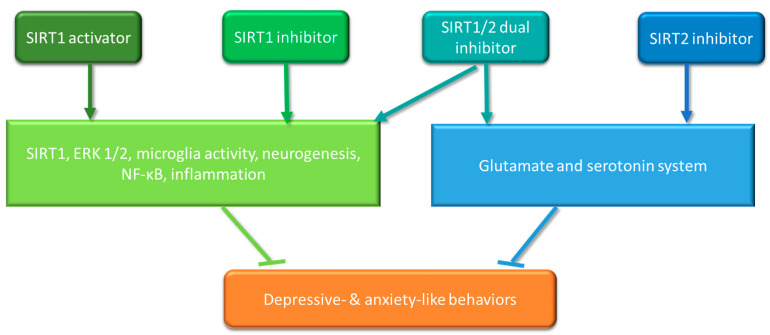
Pathways through which SIRT1, SIRT2 modulators could potentially influence depressive- and anxiety-like behaviours.

Resveratrol has been applied as a potent natural SIRT1 activator in numerous studies [52]. However, the effect of resveratrol on mood disorders is still controversial. A study conducted on stressed mice indicated that resveratrol intraperitoneal (i.p.) treatment can prevent anxiety and depression via SIRT1 activation and downstream extracellular signal-regulated kinases (ERK1/2), which have been reported as participating in a pro-depressive mechanism [53,54]. Resveratrol (i.p.) can also alleviate depressive-like behaviours and reduce microglia activation and maintain neurogenesis via SIRT1 activation and down-regulating NF-κB in lipopolysaccharide (LPS)-induced mouse models [55]. Conversely, it has also been reported that resveratrol treatment via bilateral infusion into the nucleus accumbens can induce depressive-like behaviours in chronic social defeat stress-induced mouse models [56]. Another SIRT1 activator, SRT2104, administered via bilateral infusion into the hippocampus, has also demonstrated similar protective effects against depressive-like behaviours by moderating neuroinflammation in chronic unpredictable mild-stress-induced mice [57]. Kim’s study showed that the SIRT1 inhibitor, EX527, protects stressed mice from depression and anxiety [56], but, in contrast, EX527 treatment via bilateral injection into the hippocampus led to depressive-like behaviours in chronic ultra-mild-stress-induced mice [53]. The SIRT1/2 dual inhibitor sirtinol has been shown to protect stress-mediated rat models from disorders of mood and memory function through SIRT1, ERK1/2 and the downstream Bcl-2 pathway, which is associated with apoptosis regulation [58]. Another SIRT2 inhibitor 33i has been indicated to have an antidepressant-like effect on chronic mild-stress-induced mouse models. Erburu’s studies showed that 33i (i.p.) prevents mood disorder via the modulating neurotransmitters glutamate and serotonin in the prefrontal cortex [59], and co-treatment with another deacetylase inhibitor MC1568 increased synaptic plasticity and helped to build neuronal adaption in the prefrontal cortex [60]. Overall, positive results have been shown in the limited available in vivo studies (Table 2). These studies of depressive-like behaviours in animals applied different triggers of depression (chronic stress or LPS) and different methods of drug administration (intracerebral injection or intraperitoneal injection). Pathways through which SIRT1, SIRT2 modulators could potentially influence depressive- and anxiety-like behaviours are shown in Figure 1. However, what has yet to be further elucidated is whether the anti-inflammatory effects contribute to the SIRT1 and SIRT2 modulators’ protection in the depression models and other mechanisms involved in symptoms or disorders. A further limitation of in vivo models is that they predominantly use male animals, whereas it is well-recognised that sex differences are an important factor in the manifestation and treatment of depression [61,62,63]. Therefore, in order to fully understand the role of SIRT1 and SIRT2 modulators, and potential treatment guidelines in the clinical setting, there needs to be consideration of the impact of sex in animal models of depression. 

## 5. Outlook of the Treatment Strategy for Depression

In this article, we have highlighted SIRT1 and SIRT2 enzymes’ roles in the pathophysiology of depression and the potential for their modulators to be used for their anti-inflammatory effects as an adjunct to current treatments for depression. Polymorphisms of SIRT1 and SIRT2 are associated with the risk of development of depression in multiple case-control studies. This could be due to how the expression of SIRT1 or SIRT2 is affected, and the subsequent up-regulating and/or down-regulating of activities of SIRT1 and SIRT2 can further influence the inflammatory status in depression patients. Multiple in vitro and in vivo studies have provided further supporting evidence, but consistent and accurate experiment models and broader drug screening are needed in future pre-clinical studies. Moreover, in the future, more efficient, stable and safer modulators need to be developed and their therapeutic effects assessed in depression-related clinical trials. Furthermore, because the aetiology of depression is multifactorial, including neuroinflammation, the impact of SIRT1 or SIRT2 modulators should be assessed when combined with established antidepressants. 

With the growing understanding of inflammation in depression, a phase-specific neuroimmune model has been proposed, such that anti-inflammatory medication could be tailored according to patients’ dynamic immune profiles in different clinical phases of depression, such as subsyndromal, acute and post-acute status [64]. Moreover, biomarkers such as T cell dysfunction, glial cell dysfunction, oxidative stress and anti-inflammatory cytokines, as well as pro-inflammatory cytokines, should be included when considering a patient’s inflammatory status [64]. The use of such biomarkers would help to more accurately ascertain the therapeutic effects of SIRT1 and SIRT2 modulators in different phases of depression, leading to more personalized treatment. Further investigation of the roles of SIRT1 and SIRT2 SNPs also might help target populations that are genetically susceptible to inflammation and subsequent depression symptoms or disorders.

## Figures and Tables

**Table 1:** A summary of single-nucleotide polymorphism (SNP) studies of SIRT1 and SIRT2 in the depression population.

Single-Nucleotide Polymorphism (SNP)	Population	Study Type	Subject Number	Impact
rs1245800 (SIRT1)	Chinese women with major depressive disorder (MDD)	Genome-wide association study (GWAS)	9000	SNP rs1245800 contributes to the risk of MDD [38].
rs1245800	Chinese Han population	GWAS	4855	SNP rs1245800 contributes to reduced SIRT1 expression and cerebellar grey matter volume [39].
rs1245800 and rs4746720 (SIRT1)	Japanese women	GWAS	1538	SNP rs1245800 and rs4746720 associate with suicide. No association with SIRT1 expression in the dorsolateral prefrontal cortex [40].
rs10997875 (SIRT1)	Japanese population	Case-control study	1221	SNP rs10997875 associates with MDD [41].
rs10410544 (SIRT2)	Alzheimer’s disease (AD) patients in European populations	Case-control study	1172	T/T SNP genotype might exert protection against depressive symptoms in AD [42].
rs2873703, rs4801933 (SIRT2)	Chinese women undergoing caesarean section	Case-control study	368	T/T genotype SNPs are correlated with the onset of postpartum depressive symptoms [43].

**Table 2:** A summary of findings of SIRT1 and SIRT2 modulators in depression in vivo models.

SIRT1/SIRT2 Modulators	Depressive-Like In Vivo Models	Administration Route	Results
Resveratrol (SIRT1 activator)	Adult male BALB mice + chronic ultra-mild stress	Intraperitoneal (i.p.) injection	Depression-like and anxiety-like behaviours ↓, SIRT1 activation ↑, phosphorylation of ERK1/2 ↑ [53]. *
	Adult male C57/BL6 mice + LPS challenge	i.p.	Depressive-like behaviours ↓, microglia activation ↓, neurogenesis ↑, SIRT1 ↑, NF-κB ↓ [55].
	Adult male C57/BL6 mice + chronic social defeat stress	Bilateral infusion into nucleus accumbens	Depressive- and anxiety-like behaviours ↑ [56].
SRT2104 (SIRT1 activator)	Adult male C57BL/6 mice + chronic unpredictable mild stress	Bilateral infusion into hippocampus	Depressive-like behaviour ↓, IL-6, IL-1β and iNOS ↓, IL-10, TNF-β and Abrignase1 ↑, microglia polarization ↓ via GSK3β/PTEN signalling pathway [57].
	Adult male BALB mice + repeated restraint stress	Bilateral injection into dentate gyrus	Social interaction ↑, sucrose preference ↑, phosphorylation of ERK1/2 ↑ [53].
EX527 (SIRT1 inhibitor)	Adult male C57/BL6 mice + chronic social defeat stress	Bilateral infusion into nucleus accumbens	Depressive- and anxiety-like behaviours ↓ [56].
	Adult male BALB mice + chronic ultra-mild stress	Bilateral injection into hippocampus	Social interaction ↓, latency to feed ↑ [53].
Sirtinol (SIRT1/2 dual inhibitor)	Adult male BALB mice + chronic ultra-mild stress	Bilateral injection into dentate gyrus	Social interaction ↓, latency to feed ↑, immobility time ↑ [53].
	Adult male Wistar rats + chronic variable stress	Infusion to dentate gyrus	Development of anhedonia ↓, stress-induced deficits in spatial memory ↓, ERK1/2 activity, Bcl-2 expression and histone acetylation ↑ [58].
33i (SIRT2 inhibitor)	Adult male C57BL/6J mice + chronic mild stress	i.p.	Stress-induced anhedonia and social avoidance ↓ via regulating glutamate and serotonin system in the prefrontal cortex [59].

* ↑ = an increase, ↓ = a decrease.

## References

[1] Murray C.J.L., Lopez A.D. (1996). A comprehensive assessment of mortality and disability from diseases, injuries, and risk factors in 1990 and projected to 2020. The Global Burden of Disease.

[2] World Health Organization Depression Key Facts. https://www.who.int/news-room/fact-sheets/detail/depression.

[3] Roy A., Campbell M.K. (2013). A unifying framework for depression: Bridging the major biological and psychosocial theories through stress. Clin. Investig. Med..

[4] Kohler O., Krogh J., Mors O., Benros M.E. (2016). Inflammation in Depression and the Potential for Anti-Inflammatory Treatment. Curr. Neuropharmacol..

[5] Loonen A.J.M., Ivanova S.A. (2016). Circuits Regulating Pleasure and Happiness—Mechanisms of Depression. Front. Hum. Neurosci..

[6] Uddin M., Koenen K.C., Aiello A.E., Wildman D.E., Santos R.D.L., Galea S. (2010). Epigenetic and inflammatory marker profiles associated with depression in a community-based epidemiologic sample. Psychol. Med..

[7] Schiepers O.J., Wichers M.C., Maes M. (2005). Cytokines and major depression. Prog. Neuro Psychopharmacol. Biol. Psychiatry.

[8] Miller A.H., Raison C.L. (2016). The role of inflammation in depression: From evolutionary imperative to modern treatment target. Nat. Rev. Immunol..

[9] Penn E., Tracy D.K. (2012). The drugs don’t work? antidepressants and the current and future pharmacological management of depression. Ther. Adv. Psychopharmacol..

[10] National Institute of Mental Health (2006). Sequenced Treatment Alternatives to Relieve Depression (STAR*D) Study.

[11] Trivedi M.H., Rush A.J., Wisniewski S.R., Nierenberg A.A., Warden D., Ritz L., Norquist G., Howland R.H., Lebowitz B., McGrath P.J. (2006). Evaluation of Outcomes With Citalopram for Depression Using Measurement-Based Care in STAR*D: Implications for Clinical Practice. Am. J. Psychiatry.

[12] Gaynes B.N., Warden D., Trivedi M.H., Wisniewski S.R., Fava M., Rush A.J. (2009). What did STAR*D teach us? Results from a large-scale, practical, clinical trial for patients with depression. Psychiatr. Serv..

[13] Rush A.J., Trivedi M.H., Wisniewski S.R., Stewart J.W., Nierenberg A.A., Thase M.E., Ritz L., Biggs M.M., Warden D., Luther J.F. (2006). Bupropion-SR, Sertraline, or Venlafaxine-XR after Failure of SSRIs for Depression. N. Engl. J. Med..

[14] Trivedi M.H., Fava M., Wisniewski S.R., Thase M.E., Quitkin F., Warden D., Ritz L., Nierenberg A.A., Lebowitz B.D., Biggs M.M. (2006). Medication Augmentation after the Failure of SSRIs for Depression. N. Engl. J. Med..

[15] Thase M.E., Friedman E.S., Biggs M.M., Wisniewski S.R., Trivedi M.H., Luther J.F., Fava M., Nierenberg A.A., McGrath P.J., Warden D. (2007). Cognitive therapy versus medication in augmentation and switch strategies as second-step treatments: A STAR*D report. Am. J. Psychiatry.

[16] Fava M., Rush A.J., Wisniewski S.R., Nierenberg A.A., Alpert J.E., McGrath P.J., Thase M.E., Warden D., Biggs M., Luther J.F. (2006). A comparison of mirtazapine and nortriptyline following two consecutive failed medication treatments for depressed outpatients: A STAR*D report. Am. J. Psychiatry.

[17] Nierenberg A.A., Fava M., Trivedi M.H., Wisniewski S.R., Thase M.E., McGrath P.J., Alpert J.E., Warden D., Luther J.F., Niederehe G. (2006). A Comparison of Lithium and T3Augmentation Following Two Failed Medication Treatments for Depression: A STAR*D Report. Am. J. Psychiatry.

[18] McGrath P.J., Stewart J.W., Fava M., Trivedi M.H., Wisniewski S.R., Nierenberg A.A., Thase M.E., Davis L., Biggs M.M., Shores-Wilson K. (2006). Tranylcypromine Versus Venlafaxine Plus Mirtazapine Following Three Failed Antidepressant Medication Trials for Depression: A STAR*D Report. Am. J. Psychiatry.

[19] Huynh N.N., McIntyre R.S. (2008). What are the implications of the STAR*D trial for primary care? a review and synthesis. Prim. Care Companion J. Clin. Psychiatry.

[20] Landry J., Sutton A., Tafrov S.T., Heller R.C., Stebbins J., Pillus L., Sternglanz R. (2000). The silencing protein SIR2 and its homologs are NAD-dependent protein deacetylases. Proc. Natl. Acad. Sci. USA.

[21] Jiang Y., Liu J., Chen D., Yan L., Zheng W. (2017). Sirtuin Inhibition: Strategies, Inhibitors, and Therapeutic Potential. Trends Pharmacol. Sci..

[22] Lu G., Li J., Zhang H., Zhao X., Yan L.-J., Yang X. (2018). Role and Possible Mechanisms of Sirt1 in Depression. Oxidative Med. Cell. Longev..

[23] Brites D., Fernandes A. (2015). Neuroinflammation and Depression: Microglia Activation, Extracellular Microvesicles and microRNA Dysregulation. Front. Cell. Neurosci..

[24] Xie J., Zhang X., Zhang L. (2013). Negative regulation of inflammation by SIRT. Pharmacol. Res..

[25] Paraíso A.F., Mendes K.L., Santos S.H.S. (2013). Brain Activation of SIRT1: Role in Neuropathology. Mol. Neurobiol..

[26] Gan L., Mucke L. (2008). Paths of Convergence: Sirtuins in Aging and Neurodegeneration. Neuron.

[27] He M., Chiang H.-H., Luo H., Zheng Z., Qiao Q., Wang L., Tan M., Ohkubo R., Mu W.-C., Zhao S. (2020). An Acetylation Switch of the NLRP3 Inflammasome Regulates Aging-Associated Chronic Inflammation and Insulin Resistance. Cell Metab..

[28] Buechler N., Wang X., Yoza B.K., McCall C.E., Vachharajani V. (2017). Sirtuin 2 Regulates Microvascular Inflammation during Sepsis. J. Immunol. Res..

[29] Wang B., Zhang Y., Cao W., Wei X., Chen J., Ying W. (2016). SIRT2 plays significant roles in lpopolysaccharides-induced neuroinflammation and brain injury in mice. Neurochem. Res..

[30] Lavu S., Boss O., Elliott P.J., Lambert P.D. (2008). Sirtuins—Novel therapeutic targets to treat age-associated diseases. Nat. Rev. Drug Discov..

[31] Fu G., Chen S., Liang L., Li X., Tang P., Rao X., Pan M., Xu X., Li Y., Yao Y. (2021). SIRT1 inhibitors mitigate radiation-induced GI syndrome by enhancing intestinal-stem-cell survival. Cancer Lett..

[32] Frazzi R. (2018). SIRT1 in Secretory Organ Cancer. Front. Endocrinol..

[33] Wang T.-W., Chern E., Hsu C.-W., Tseng K.-C., Chao H.-M. (2020). SIRT1-Mediated Expression of CD24 and Epigenetic Suppression of Novel Tumor Suppressor miR-1185-1 Increases Colorectal Cancer Stemness. Cancer Res..

[34] van der Meer A.J., Scicluna B.P., Moerland P.D., Lin J., Jacobson E.W., Vlasuk G.P., van der Poll T. (2015). The Selective Sirtuin 1 Activator SRT2104 Reduces Endotoxin-Induced Cytokine Release and Coagulation Activation in Humans. Crit. Care Med..

[35] Krueger J.G., Suárez-Fariñas M., Cueto I., Khacherian A., Matheson R., Parish L.C., Leonardi C., Shortino D., Gupta A., Haddad J. (2015). A Randomized, Placebo-Controlled Study of SRT2104, a SIRT1 Activator, in Patients with Moderate to Severe Psoriasis. PLoS ONE.

[36] Dean J., Keshavan M. (2017). The neurobiology of depression: An integrated view. Asian J. Psychiatry.

[37] Shadrina M., Bondarenko E.A., Slominsky P.A. (2018). Genetics Factors in Major Depression Disease. Front. Psychiatry.

[38] Cai N., Bigdeli T.B., Kretzschmar W., Li Y., Liang J., Song L. (2015). Sparse whole-genome sequencing identifies two loci for major depressive disorder. Nature.

[39] Liu W., Yan H., Zhou D., Cai X., Zhang Y., Li S., Zhou D.-S., Li X., Zhang C., Sun Y. (2019). The depression GWAS risk allele predicts smaller cerebellar gray matter volume and reduced SIRT1 mRNA expression in Chinese population. Transl. Psychiatry.

[40] Hirata T., Otsuka I., Okazaki S., Mouri K., Horai T., Boku S., Takahashi M., Ueno Y., Sora I., Shirakawa O. (2019). Major depressive disorder-associated SIRT1 locus affects the risk for suicide in women after middle age. Psychiatry Res..

[41] Kishi T., Yoshimura R., Kitajima T., Okochi T., Okumura T., Tsunoka T., Yamanouchi Y., Kinoshita Y., Kawashima K., Fukuo Y. (2010). SIRT1 gene is associated with major depressive disorder in the Japanese population. J. Affect. Disord..

[42] Porcelli S., Salfi R., Politis A., Atti A.R., Albani D., Chierchia A., Polito L., Zisaki A., Piperi C., Liappas I. (2013). Association between Sirtuin 2 gene rs10410544 polymorphism and depression in Alzheimer’s disease in two independent European samples. J. Neural Transm..

[43] Luo S.-C., Duan K.-M., Fang C., Li D.-Y., Zheng S.-S., Yang S.-Q., Yang S.-T., Yang M., Zhang L.-B., Wang S.-Y. (2020). Correlations Between SIRT Genetic Polymorphisms and Postpartum Depressive Symptoms in Chinese Parturients Who Had Undergone Cesarean Section. Neuropsychiatr. Dis. Treat..

[44] Himmerich H., Patsalos O., Lichtblau N., Ibrahim M.A.A., Dalton B. (2019). Cytokine Research in Depression: Principles, Challenges, and Open Questions. Front. Psychiatry.

[45] Coutinho D.D.S., Pacheco M.T., Frozza R.L., Bernardi A. (2018). Anti-Inflammatory Effects of Resveratrol: Mechanistic Insights. Int. J. Mol. Sci..

[46] Hoffmann E., Wald J., Lavu S., Roberts J., Beaumont C., Haddad J., Elliott P., Westphal C., Jacobson E. (2012). Pharmacokinetics and tolerability of SRT2104, a first-in-class small molecule activator of SIRT1, after single and repeated oral administration in man. Br. J. Clin. Pharmacol..

[47] Donmez G., Outeiro T.F. (2013). SIRT1 and SIRT2: Emerging targets in neurodegeneration. EMBO Mol. Med..

[48] Yuan F., Xu Z.M., Lu L.Y., Nie H., Ding J., Ying W.H., Tian H.L. (2016). SIRT2 inhibition exacerbates neuroinflammation and blood-brain barrier disruption in experimental traumatic brain injury by enhancing NF-κB p65 acetylation and activation. J. Neurochem..

[49] Romeo-Guitart D., Leiva-Rodríguez T., Espinosa-Alcantud M., Sima N., Vaquero A., Martín H.D.-, Ruano D., Casas C. (2018). SIRT1 activation with neuroheal is neuroprotective but SIRT2 inhibition with AK7 is detrimental for disconnected motoneurons. Cell Death Dis..

[50] Lee Y.G., Reader B.F., Herman D., Streicher A., Englert J.A., Ziegler M., Chung S., Karpurapu M., Park G.Y., Christman J.W. (2019). Sirtuin 2 enhances allergic asthmatic inflammation. JCI Insight.

[51] Orecchia A., Scarponi C., Di Felice F., Cesarini E., Avitabile S., Mai A., Mauro M.L., Sirri V., Zambruno G., Albanesi C. (2011). Sirtinol treatment reduces inflammation in human dermal microvascular endothelial cells. PLoS ONE.

[52] Dai H., Sinclair D.A., Ellis J.L., Steegborn C. (2018). Sirtuin activators and inhibitors: Promises, achievements, and challenges. Pharmacol. Ther..

[53] Abe-Higuchi N., Uchida S., Yamagata H., Higuchi F., Hobara T., Hara K., Kobayashi A., Watanabe Y. (2016). Hippocampal Sirtuin 1 Signaling Mediates Depression-like Behavior. Biol. Psychiatry.

[54] Borges G., Berrocoso E., Mico J.A., Neto F. (2015). ERK1/2: Function, signaling and implication in pain and pain-related anxio-depressive disorders. Prog. Neuro Psychopharmacol. Biol. Psychiatry.

[55] Liu L., Zhang Q., Cai Y., Sun D., He X., Wang L., Yu D., Li X., Xiong X., Xu H. (2016). Resveratrol counteracts lipopolysaccharide-induced depressive-like behaviors via enhanced hippocampal neurogenesis. Oncotarget.

[56] Kim H.-D., Hesterman J., Call T., Magazu S., Keeley E., Armenta K., Kronman H., Neve R.L., Nestler E.J., Ferguson D. (2016). SIRT1 Mediates Depression-Like Behaviors in the Nucleus Accumbens. J. Neurosci..

[57] Duan C.-M., Zhang J.-R., Wan T.-F., Wang Y., Chen H.-S., Liu L. (2020). SRT2104 attenuates chronic unpredictable mild stress-induced depressive-like behaviors and imbalance between microglial M1 and M2 phenotypes in the mice. Behav. Brain Res..

[58] Ferland C.L., Hawley W.R., Puckett R.E., Wineberg K., Lubin F.D., Dohanich G.P., Schrader L.A. (2013). Sirtuin Activity in Dentate Gyrus Contributes to Chronic Stress-Induced Behavior and Extracellular Signal-Regulated Protein Kinases 1 and 2 Cascade Changes in the Hippocampus. Biol. Psychiatry.

[59] Erburu M., Muñoz-Cobo I., Diaz-Perdigon T., Mellini P., Suzuki T., Puerta E., Tordera R.M. (2017). SIRT2 inhibition modulate glutamate and serotonin systems in the prefrontal cortex and induces antidepressant-like action. Neuropharmacology.

[60] Erburu M., Muñoz-Cobo I., Domínguez-Andrés J., Beltran E., Suzuki T., Mai A., Valente S., Puerta E., Tordera R. (2015). Chronic stress and antidepressant induced changes in Hdac5 and Sirt2 affect synaptic plasticity. Eur. Neuropsychopharmacol..

[61] Palanza P. (2001). Animal models of anxiety and depression: How are females different?. Neurosci. Biobehav. Rev..

[62] Eid R.S., Gobinath A.R., Galea L.A. (2019). Sex differences in depression: Insights from clinical and preclinical studies. Prog. Neurobiol..

[63] Lei Y., Wang J., Wang D., Li C., Liu B., Fang X., You J., Guo M., Lu X.-Y. (2020). SIRT1 in forebrain excitatory neurons produces sexually dimorphic effects on depression-related behaviors and modulates neuronal excitability and synaptic transmission in the medial prefrontal cortex. Mol. Psychiatry.

[64] Eyre H., Stuart M., Baune B. (2014). A phase-specific neuroimmune model of clinical depression. Prog. Neuro-Psychopharmacol. Biol. Psychiatry.

